# Evolution of artificial intelligence in healthcare: a 30-year bibliometric study

**DOI:** 10.3389/fmed.2024.1505692

**Published:** 2025-01-15

**Authors:** Yaojue Xie, Yuansheng Zhai, Guihua Lu

**Affiliations:** ^1^Yangjiang Bainian Yanshen Medical Technology Co., Ltd., Yangjiang, China; ^2^Department of Cardiology, Heart Center, First Affiliated Hospital, Sun Yat-sen University, Guangzhou, China; ^3^NHC Key Laboratory of Assisted Circulation (Sun Yat-sen University), Guangzhou, China

**Keywords:** artificial intelligence, health care, medicine, ChatGPT, bibliometric study

## Abstract

**Introduction:**

In recent years, the development of artificial intelligence (AI) technologies, including machine learning, deep learning, and large language models, has significantly supported clinical work. Concurrently, the integration of artificial intelligence with the medical field has garnered increasing attention from medical experts. This study undertakes a dynamic and longitudinal bibliometric analysis of AI publications within the healthcare sector over the past three decades to investigate the current status and trends of the fusion between medicine and artificial intelligence.

**Methods:**

Following a search on the Web of Science, researchers retrieved all reviews and original articles concerning artificial intelligence in healthcare published between January 1993 and December 2023. The analysis employed Bibliometrix, Biblioshiny, and Microsoft Excel, incorporating the bibliometrix R package for data mining and analysis, and visualized the observed trends in bibliometrics.

**Results:**

A total of 22,950 documents were collected in this study. From 1993 to 2023, there was a discernible upward trajectory in scientific output within bibliometrics. The United States and China emerged as primary contributors to medical artificial intelligence research, with Harvard University leading in publication volume among institutions. Notably, the rapid expansion of emerging topics such as COVID-19 and new drug discovery in recent years is noteworthy. Furthermore, the top five most cited papers in 2023 were all pertinent to the theme of ChatGPT.

**Conclusion:**

This study reveals a sustained explosive growth trend in AI technologies within the healthcare sector in recent years, with increasingly profound applications in medicine. Additionally, medical artificial intelligence research is dynamically evolving with the advent of new technologies. Moving forward, concerted efforts to bolster international collaboration and enhance comprehension and utilization of AI technologies are imperative for fostering novel innovations in healthcare.

## 1 Introduction

With the continuous advancement of science and technology, particularly in the fields of computer science, data processing, and machine learning, the application of artificial intelligence (AI) technology in healthcare has become increasingly widespread (1–3). Current research has demonstrated that the integration of healthcare and AI enhances patient care, improves efficiency, and reduces costs in the healthcare industry, leading to smarter, faster, and more efficient healthcare systems (4–7). This optimization spans from diagnosis to treatment planning, driving advancements in disease prediction, diagnosis, and therapeutic interventions, thereby providing substantial benefits for both patients and healthcare providers (8–10).

Artificial intelligence has extensive applications in medicine, including risk assessment, triage, diagnosis, follow-up management, drug development, and therapies (11, 12). In risk assessment, AI algorithms are utilized to analyze patient data and identify individuals at high risk for developing specific conditions, allowing for early intervention and prevention strategies (13). Primary care, as the frontline of healthcare, plays a vital role in prevention, chronic disease management, and personalized guidance. AI-powered tools in primary care can assist in early screening for conditions such as diabetes, hypertension, and mental health disorders, enabling timely management and reducing the progression of these diseases (14). For chronic disease management, AI systems can provide personalized recommendations based on real-time patient data, helping healthcare providers create tailored lifestyle and treatment plans (15).

ChatGPT is a sophisticated language model designed using deep learning algorithms to produce responses that closely resemble human conversation. As part of the Generative Pre-trained Transformer (GPT) series developed by OpenAI, it stands out as one of the largest and most accessible language models currently available (16). Utilizing a vast repository of textual data, ChatGPT excels in capturing the intricacies and subtleties of human language, allowing it to deliver highly relevant and context-aware responses across a diverse array of prompts. More recently, its functionality has expanded beyond text generation to include the creation of images and videos, significantly enhancing its multimodal capabilities and widening its applicability in areas such as healthcare, education, and creative industries (17). With their rapid development, these models are poised to take on a growing role in medical research, from facilitating systematic reviews to advancing personalized treatment approaches. As they continue to evolve, large language models have the potential to drive medical innovation forward, improving patient outcomes and enabling data-driven, precise decision-making in various areas of healthcare (18–20).

To better understand the evolution and impact of AI in healthcare, bibliometric studies are essential for systematically mapping the research landscape and identifying emerging trends. This study aims to meticulously retrieve relevant literature from the Web of Science Core Collection database (WoSCC) from January 1, 1993, to December 31, 2023. Through quantitative and visual network analyses encompassing various parameters such as authors, institutions, countries/regions, and keywords, this analysis is expected to assist researchers in gaining comprehensive insights into AI-related research in healthcare and predicting future patterns and trends.

## 2 Database and methods

### 2.1 Bibliometric database

The selection of the Web of Science Core Collection (WoSCC) was based on its ability to provide comprehensive data meeting the needs of bibliometric software, and its reputation as a prominent database in this research domain. Consequently, the bibliometric analysis yielded invaluable insights into the prevailing status of AI research in healthcare up to December 2023. Since the data are derived from publicly available databases, ethical approval for this study was considered unnecessary. To avoid bias, we decided not to include such specific algorithmic terms in the search.

### 2.2 Eligibility criteria

This study systematically identified relevant research by focusing on two primary categories: (1) AI technologies and (2) healthcare and medicine. The selection of search keywords was informed by an extensive preliminary literature review and consultations with researchers and domain experts. Keywords for AI technologies were chosen to represent a wide range of approaches and advancements in the field, while healthcare-related keywords ensured relevance to medical and healthcare domains. The finalized search formula was as follows:

[TI = ("artificial intelligence") OR TI = ("data learning") OR TI = ("machine learning") OR TI = ("expert systems") OR TI = ("fuzzy logic") OR TI = ("computer vision") OR TI = ("automatic programming") OR TI = ("speech understanding") OR TI = ("autonomous robots") OR TI = ("intelligent tutoring") OR TI = ("intelligent agents") OR TI = ("neural network") OR TI = ("voice recognition") OR TI = ("text mining") OR TI = ("electronic health record") OR TI = ("ChatGPT") OR TI = ("large language models")] AND [TS = (health) OR TS = (healthcare) OR TS = (medicine) OR TS = (mental health) OR TS = (behavioral health)].

To ensure high-quality and peer-reviewed content, the study included only publications classified as "article" or "review article." This excluded other document types such as conference abstracts, letters, expert opinions, editorial materials, corrections, retractions, and conference papers, which were considered less representative of the core focus of the study.

The study assessed publications within the period from January 1, 1993, to December 31, 2023. This 30-year timeframe was selected to comprehensively capture the evolution of AI technologies and their applications in healthcare and medicine. The analysis was restricted to English-language publications to ensure consistency in the evaluation and accessibility of results.

By adopting these eligibility criteria, this study aimed to identify and analyze literature trends, emerging technologies, and interdisciplinary applications of AI in healthcare, while maintaining a focus on reliability and academic rigor.

### 2.3 Data analysis

Bibliometrix, Biblioshiny, and Microsoft Excel are used for data analysis and visualization of AI in health care article studies. Data were presented via descriptive statistics. Inferential bibliometric analyses included clustering of the selected parameters of keywords, keyword plus, titles, and abstracts; Bradford’s law to evaluate core journals, and the Sankey diagrams to evaluate the flows between research themes over time. They can visualize the research results and has unique advantages in clustering technology and map display.

## 3 Results

### 3.1 Overview

**FIGURE 1:**
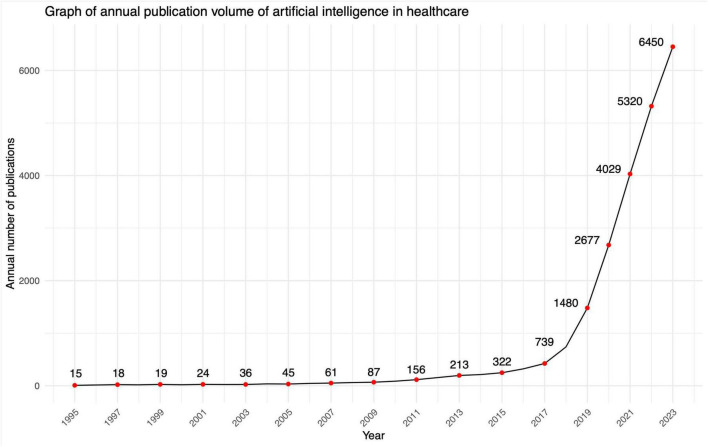
Annual publication volume of artificial intelligence in healthcare.

A total of 22,950 documents were collected from 5,024 sources, with these sources specifically referring to academic journals. The dataset revealed an average annual growth rate of 26.97%, alongside an average document age of 3.41 years and an average of 17 citations per document. From 1993 to 2023, a notable upward trend has been observed in the annual scientific output within the field of bibliometrics. In the initial years, such as 1993 and 1994, the volume of scientific publications remained comparatively modest, with only 5 and 11 articles, respectively. Subsequent to 2010, this growth trajectory accelerated further, characterized by a marked escalation in the annual growth rate. Notably, the period spanning from 2019 to 2023 witnessed a particularly remarkable expansion in scientific productivity, yielding 1480, 2677, 4029, 5320, and 6450 articles respectively (Figure 1).

### 3.2 Countries

**FIGURE 2:**
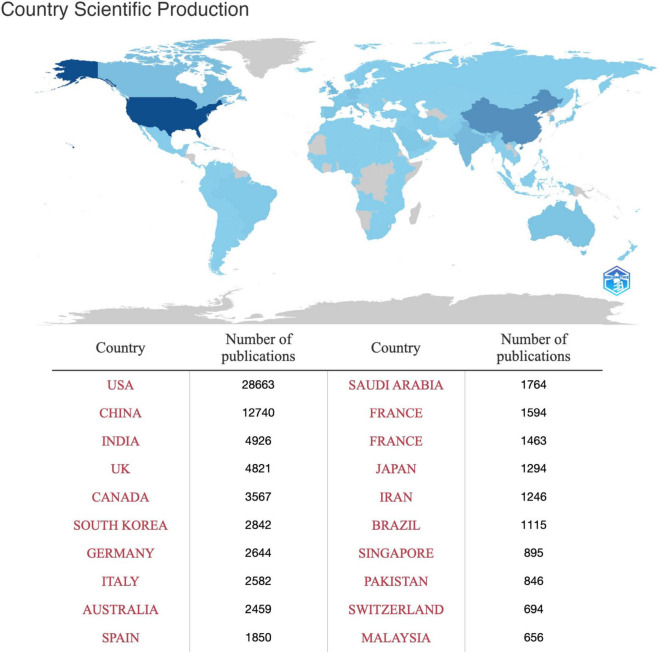
Country-specific production. The color intensity is proportional to the number of documents. Higher blue intensity refers to greater number of documents (dark blue = high productivity; gray = no documents).

The scientific production of various countries offers valuable insights into their respective contributions to research. The United States leads with an impressive count of 28,663 articles, highlighting its significant influence and productivity within the scientific community. Following closely, China presents 12,740 articles, indicating its rapid growth and importance in scientific research. India ranks third, contributing 4,926 articles and establishing itself as a significant player in scientific inquiry. The United Kingdom maintains a robust presence with 4,821 articles, showcasing its active involvement and contributions to scientific endeavors. Canada follows suit with 3,567 articles, making substantial contributions to the global scientific landscape (Figure 2).

**FIGURE 3:**
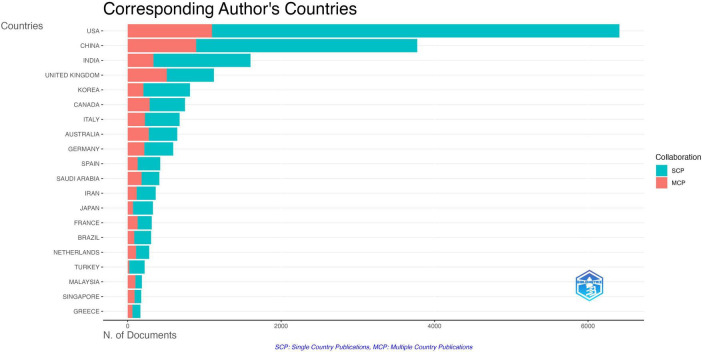
Corresponding author’s countries. Countries of the top 20 most relevant corresponding authors’ articles of artificial intelligence in health care in the Web of Science Core Collection. SCP, Single Country Publications; MCP, Multiple Country Publications.

The article counts and collaboration patterns based on the authors’ countries reveal interesting trends. The United States leads in article count, having published a total of 6,409 articles, comprising 5,310 single corresponding author papers (SCP) and 1,099 multiple corresponding author papers (MCP). Despite its high MCP count, the US exhibits a comparatively lower MCP-to-total articles ratio of 0.171 compared to other countries. China closely follows with 3,774 articles, consisting of 2,882 SCPs and 892 MCPs, demonstrating a higher ratio of MCPs, indicating extensive collaborative research efforts. India contributes 1601 articles, with a higher proportion of SCPs than MCPs, aligning with its lower MCP frequency of 0.070. It is worth noting that contributions from the United Kingdom, South Korea, Canada, Australia, Italy, Germany, and Spain are also notable, with each country demonstrating a relatively high MCP ratio (Figure 3). These findings underscore the global distribution of research output in medical AI, highlighting diverse collaborative patterns among researchers from different countries.

### 3.3 Sources

The top 20 research institutions in the field of medical AI, as indicated by publication volume, exhibit a concentration of academic powerhouses renowned for their research contributions. Leading the pack is Harvard University, with an impressive 1,690 articles, followed closely by the University of California System with 1,180 articles. Harvard Medical School, a prestigious institution affiliated with Harvard University, demonstrates a strong presence with 802 articles. The University of Toronto and the University of Pennsylvania also showcase significant engagement in this field, with 809 and 635 articles respectively. Other notable institutions include the University of Michigan, University of London, Stanford University, University System of Ohio, and the University of California San Francisco, each contributing substantially to the body of literature in medical AI.

In the research publications, PLOS ONE leads with a publication count of 370 articles, possibly owing to its open access policy and broad research scope. Following closely is IEEE ACCESS, with 360 articles, showcasing the diversity and breadth of the field. SCIENTIFIC REPORTS ranks third with 358 articles, highlighting the intersection of medical AI. The JOURNAL OF MEDICAL INTERNET RESEARCH, with 347 articles, likely covers topics related to sensor technology and its applications in medical AI. Additionally, the JOURNAL OF THE AMERICAN MEDICAL INFORMATICS ASSOCIATION and SENSORS hold significant influence in the medical informatics field with 347 and 338 articles, respectively. The publication volume of these journals reflects the continued growth and interdisciplinary nature of the medical artificial intelligence field. Other journals such as APPLIED SCIENCES-BASEL, JMIR MEDICAL INFORMATICS, and BMC MEDICAL INFORMATICS AND DECISION MAKING also make substantial contributions, demonstrating the diversity and activity within the field of medical artificial intelligence.

In 2023, bibliometric analysis of medical artificial intelligence research unveiled several highly cited global publications. Leading the list is the review article titled "ChatGPT Utility in Healthcare Education, Research, and Practice: Systematic Review on the Promising Perspectives and Valid Concerns," published in the journal "Healthcare," which garnered 332 citations (21). Following closely is the Original Investigation article "Comparing Physician and Artificial Intelligence Chatbot Responses to Patient Questions Posted to a Public Social Media Forum," published in JAMA Internal Medicine, with 238 citations (22). Additionally, a review from Cureus titled "Artificial Hallucinations in ChatGPT: Implications in Scientific Writing" ranks as the third most cited (23), while an article from the Journal of Medical Systems titled "Evaluating the Feasibility of ChatGPT in Healthcare: An Analysis of Multiple Clinical and Research Scenarios" holds the fourth position (24). Notably, the top five most cited publications all center around topics related to ChatGPT.

### 3.4 Documents

Table 1 presents an overview of the top ten most globally cited reviews and articles within the field of medical artificial intelligence. The most globally cited review, "Machine learning: Trends, perspectives, and prospects," authored by M. I. Jordan from the University of California and T. M. Mitchell from Carnegie Mellon University, was published in the Science journal on July 17, 2015 (25). The top three most globally cited articles in the field of medical artificial intelligence literature are as follows: Guo L’s article titled "A multi-time scale approach to remaining useful life prediction in rolling bearing," published in Neurocomputing in 2017, investigates a multi-time scale modeling approach for predicting the remaining useful life (RUL) of rolling bearings. This study contributes to the advancement of predictive maintenance techniques in industrial applications (26). The second article, authored by A. Bate and titled "A Bayesian neural network method for adverse drug reaction signal generation," was published in the European Journal of Clinical Pharmacology in 1998 and has accumulated 692 citations globally (27). This article suggests that Bayesian neural network models can effectively detect significant signals from adverse drug reaction data, particularly from the WHO Programme on International Drug Monitoring dataset. Lastly, Wang LD’s research, titled "COVID-Net: a tailored deep convolutional neural network design for the detection of COVID-19 cases from chest X-ray images," stands out with 690 citations worldwide (28).

**TABLE 1:** Top most globally cited reviews and articles.

Time	Types	Documents	DOI	TC	TC per year	Normalized TC
1993–2023	REVIEW	JORDAN MI, 2015, SCIENCE	doi: 10.1126/science.aaa841	3642	364.2	58.43
1993–2023	REVIEW	RUDIN C, 2019, NAT MACH INTELL	doi: 10.1038/s42256-019-0048-x	2430	405	60.29
1993–2023	REVIEW	TOPOL EJ, 2019, NAT MED	doi: 10.1038/s41591-018-0300-7	2146	357.67	53.24
1993–2023	REVIEW	DEO RC, 2015, CIRCULATION	doi: 10.1161/CIRCULATIONAHA.115.001593	1508	150.8	24.19
1993–2023	REVIEW	JIANG F, 2017, STROKE VASC NEUROL	doi: 10.1136/svn-2017-00010	1240	155	22.7
1993–2023	REVIEW	LEI YG, 2020, MECH SYST SIGNAL PR	doi: 10.1016/j.ymssp.2019.10658	1147	229.4	40.29
1993–2023	REVIEW	ZANG YP, 2015, MATER HORIZ	doi: 10.1039/c4mh00147	895	89.5	14.36
1993–2023	REVIEW	ZADEH LA, 2008, INFORM SCIENCES	doi: 10.1016/j.ins.2008.02.01	881	51.82	16.75
1993–2023	REVIEW	KONONENKO I, 2001, ARTIF INTELL MED	doi: 10.1016/S0933-3657(01)00077-x	823	34.29	13.66
1993–2023	REVIEW	BI WL, 2019, CA-CANCER J CLIN	doi: 10.3322/caac.2155	778	129.67	19.3
1993–2023	ARITICLE	GUO L, 2017, NEUROCOMPUTING	doi: 10.1016/j.neucom.2017.02.045	728	91	13.33
1993–2023	ARITICLE	BATE A, 1998, EUR J CLIN PHARMACOL	doi: 10.1007/s00228005046	692	25.63	10.68
1993–2023	ARITICLE	WANG LD, 2020, SCI REP-UK	doi: 10.1038/s41598-020-76550-z	690	138	24.24
1993–2023	ARITICLE	CLEGG A, 2016, AGE AGING	doi: 10.1093/aging/afw03	686	76.22	19.02
1993–2023	ARITICLE	BEN ALI J, 2015, APPL ACOUST	doi: 10.1016/j.apacoust.2014.08.01	560	56	8.98
1993–2023	ARITICLE	KANG E, 2017, MED PHYS	doi: 10.1002/mp.1234	507	63.38	9.28
1993–2023	ARITICLE	UDDIN S, 2019, BMC MED INFORM DECIS	doi: 10.1186/s12911-019-1004-8	493	82.17	12.23
1993–2023	ARITICLE	LONGONI C, 2019, J CONSUM RES	doi: 10.1093/jcr/ucz01	452	75.33	11.21
1993–2023	ARITICLE	CHEN M, 2017, IEEE ACCESS	doi: 10.1109/ACCESS.2017.269444	451	56.38	8.26
1993–2023	ARITICLE	HAN X, 2017, MED PHYS	doi: 10.1002/mp.1215	450	56.25	8.24
2023	REVIEW	SALLAM M, 2023, HEALTHCARE-BASEL	doi: 10.3390/healthcare1106088	332	166.00	125.51
2023	ARITICLE	AYERS JW, 2023, JAMA INTERN MED	doi: 10.1001/jamainternmed.2023.183	238	119.00	89.98
2023	REVIEW	ALKAISSI H, 2023, CUREUS J MED SCIENCE	doi: 10.7759/cureus.3517	205	102.50	77.50
2023	ARITICLE	CASCELLA M, 2023, J MED SYST	doi: 10.1007/s10916-023-01925-4	153	76.50	57.84
2023	REVIEW	SALVAGNO M, 2023, CRIT CARE	doi: 10.1186/s13054-023-04380-2	137	68.50	51.79

TC, total content.

### 3.5 Topics

**FIGURE 4:**
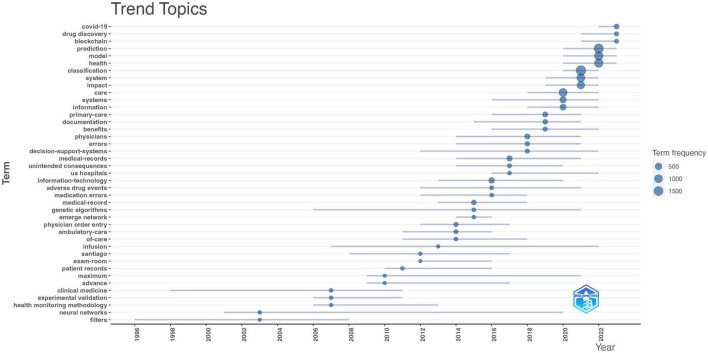
Trend topic analysis of artificial intelligence in healthcare.

From Figure 4, it can be seen that these Trend Topics were calculated using keywords plus from the literature. We observed several important themes. Among them, neural networks have received widespread attention in this field, appearing 11 times with a time span from 2001 to 2020, showing a gradually increasing trend. Clinical medicine is also one of the research hotspots, appearing 13 times, covering the period from 1998 to 2011, indicating long-term research attention. Research on medical records has also been highly regarded, appearing as many as 181 times, spanning from 2014 to 2021, and showing an increasing trend year by year, demonstrating the emphasis on medical information management. Decision support systems also play an important role in the field of medical artificial intelligence, appearing 52 times from 2012 to 2022, indicating sustained research interest. In addition, topics such as primary care, forecasting, models, and health have also received widespread attention, with research frequencies of 151, 1607, 1248, and 1230 times respectively, showing a continuous growth trend in recent years. Additionally, emerging topics such as COVID-19 and drug discovery have also attracted considerable research attention in a short period, indicating the rapid response and adaptability of the field of medical artificial intelligence to current important issues.

The bibliometric analysis of national cooperation depicted in this graph illustrates the collaborative research endeavors among different countries. The size of nodes in the graph represents the research output (number of published papers) of each country, while the connections between nodes indicate collaborative relationships, with thicker lines denoting closer cooperation. From this graph, it can be observed that China stands out as the leading contributor in artificial intelligence in health care research, demonstrating both prolific research output and frequent collaboration with other countries, thus showcasing its prominent position in international research collaboration. Additionally, countries like the United States (unlabeled), Japan, and South Africa exhibit strong inclinations toward collaboration.

### 3.6 Co-occurrences

**FIGURE 5:**
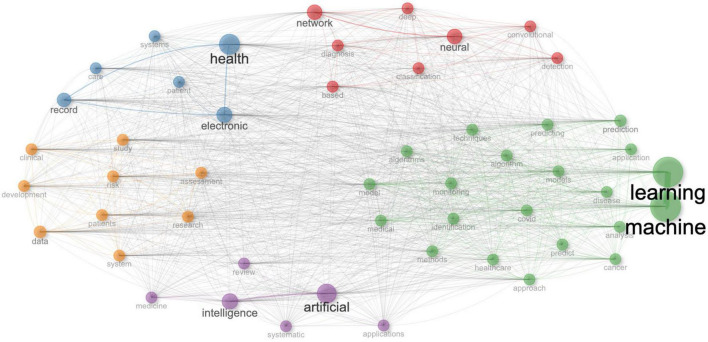
Co-occurrence network analysis of titles of artificial intelligence in healthcare.

The analysis of co-occurrence networks based on titles in bibliometrics is a method used to extract keywords from a large number of document titles and construct co-occurrence networks based on these keywords, revealing the associations and connections among documents. Our study indicates that after categorizing the extracted titles, they can be classified into five groups (Figure 5). Cluster 1 focuses on the application of neural networks in medical imaging, including terms like "neural," "network," "based," "detection," "convolutional," "deep," "diagnosis," and "classification." This c With the continuous advancement of science and technology, particularly in the fields of computer science, data processing, and machine learning, the application of artificial intelligence (AI) technology in healthcare has become increasingly widespread (1–3). Current research has demonstrated that the integration of healthcare and AI enhances patient care, improves efficiency, and reduces costs in the healthcare industry, leading to smarter, faster, and more efficient healthcare systems (4–7). This optimization spans from diagnosis to treatment planning, driving advancements in disease prediction, diagnosis, and therapeutic interventions, thereby providing substantial benefits for both patients and healthcare providers (8–10).

In 2022, OpenAI publicly introduced ChatGPT globally, marking the introduction of a new generation of powerful generative artificial intelligence tools (16, 17). This introduction has fundamentally altered the way people interact with AI technology and has sparked widespread interest and adoption across various fields, pushing the impact of AI on the medical field to new heights (18–20).

This study aims to meticulously retrieve relevant literature from the Web of Science Core Collection database (WoSCC) from January 1, 1993, to December 31, 2023. Through quantitative and visual network analyses encompassing various parameters such as authors, institutions, countries/regions, and keywords, this analysis is expected to assist researchers in gaining comprehensive insights into AI-related research in healthcare and predicting future patterns and trends.

## 4 Database and methods

### 4.1 Bibliometric database

The selection of the Web of Science Core Collection (WoSCC) was based on its ability to provide comprehensive data meeting the needs of bibliometric software, and its reputation as a prominent database in this research domain. Consequently, the bibliometric analysis yielded invaluable insights into the prevailing status of AI research in healthcare up to December 2023. Since the data are derived from publicly available databases, ethical approval for this study was considered unnecessary. Although the WoSCC predominantly includes English-language literature, it ensures high-quality and impactful sources, making it the most appropriate data source for a comprehensive, multilingual perspective. Given that the data are derived from publicly available databases, ethical approval for this study was deemed unnecessary. While PubMed is vital for biomedical and clinical research, its inclusion of non-peer-reviewed articles and conference abstracts may compromise the reliability of bibliometric analyses. Similarly, Scopus, despite its widespread use, exhibits variability in disciplinary coverage and journal quality, which could affect the precision of the analysis. Therefore, WoSCC is the most suitable database for our objectives.

### 4.2 Eligibility criteria

This study identified search keywords related to (1) AI technologies and (2) healthcare and medicine from preliminary literature reviews and consultation with researchers. Document types were limited to "article" and "review article". Conference abstracts, letters, expert views, editorial materials, corrections, retractions, and conference papers were excluded. (TI = ("artificial intelligence") OR TI = ("data learning") OR TI = ("machine learning") OR TI = ("expert systems") OR TI = ("fuzzy logic") OR TI = ("computer vision") OR TI = ("automatic programming") OR TI = ("speech understanding") OR TI = ("autonomous robots") OR TI = ("intelligent tutoring") OR TI = ("intelligent agents") OR TI = ("neural network") OR TI = ("voice recognition") OR TI = ("text mining") OR TI = ("electronic health record") OR TI = ("ChatGPT") OR TI = ("large language models")) AND (TS = (health) OR TS = (healthcare) OR TS = (medicine) OR TS = (mental health) OR TS = (behavioral health)). The assessment period for published studies was from January 1, 1993, until December 31, 2023. with language restricted to English.

### 4.3 Data analysis

Bibliometrix, Biblioshiny, and Microsoft Excel are used for data analysis and visualization of AI in health care article studies. Data were presented via descriptive statistics. Inferential bibliometric analyses included clustering of the selected parameters of keywords, keyword plus, titles, and abstracts; Bradford’s law to evaluate core journals, and the Sankey diagrams to evaluate the flows between research themes over time. They can visualize the research results and has unique advantages in clustering technology and map display.

## 5 Results

### 5.1 Overview

Following a search strategy, a total of 22,950 documents were gathered from 5,024 distinct sources. The dataset revealed an average annual growth rate of 26.97%, alongside an average document age of 3.41 years and an average of 17 citations per document. From 1993 to 2023, a notable upward trend has been observed in the annual scientific output within the field of bibliometrics. In the initial years, such as 1993 and 1994, the volume of scientific publications remained comparatively modest, with only 5 and 11 articles respectively. Subsequent to 2010, this growth trajectory accelerated further, characterized by a marked escalation in the annual growth rate. Notably, the period spanning from 2019 to 2023 witnessed a particularly remarkable expansion in scientific productivity, yielding 1480, 2677, 4029, 5320, and 6450 articles respectively (Figure 1).

### 5.2 Countries

The scientific production of various countries offers valuable insights into their respective contributions to research. The United States leads with an impressive count of 28663 articles, highlighting its significant influence and productivity within the scientific community. Following closely, China presents 12740 articles, indicating its rapid growth and importance in scientific research. India ranks third, contributing 4926 articles and establishing itself as a significant player in scientific inquiry. The United Kingdom maintains a robust presence with 4821 articles, showcasing its active involvement and contributions to scientific endeavors. Canada follows suit with 3567 articles, making substantial contributions to the global scientific landscape (Figure 2).

The article counts and collaboration patterns based on the authors’ countries reveal interesting trends. The United States leads in article count, having published a total of 6409 articles, comprising 5310 single corresponding author papers (SCP) and 1099 multiple corresponding author papers (MCP). Despite its high MCP count, the US exhibits a comparatively lower MCP-to-total articles ratio of 0.171 compared to other countries. China closely follows with 3774 articles, consisting of 2882 SCPs and 892 MCPs, demonstrating a higher ratio of MCPs, indicating extensive collaborative research efforts. India contributes 1601 articles, with a higher proportion of SCPs than MCPs, aligning with its lower MCP frequency of 0.070. It is worth noting that contributions from the United Kingdom, South Korea, Canada, Australia, Italy, Germany, and Spain are also notable, with each country demonstrating a relatively high MCP ratio (Figure 3). These findings underscore the global distribution of research output in medical AI, highlighting diverse collaborative patterns among researchers from different countries.

### 5.3 Sources

The top 20 research institutions in the field of medical AI, as indicated by publication volume, exhibit a concentration of academic powerhouses renowned for their research contributions. Leading the pack is Harvard University, with an impressive 1690 articles, followed closely by the University of California System with 1180 articles. Harvard Medical School, a prestigious institution affiliated with Harvard University, demonstrates a strong presence with 802 articles. The University of Toronto and the University of Pennsylvania also showcase significant engagement in this field, with 809 and 635 articles respectively. Other notable institutions include the University of Michigan, University of London, Stanford University, University System of Ohio, and the University of California San Francisco, each contributing substantially to the body of literature in medical AI.

In the research publications, PLOS ONE leads with a publication count of 370 articles, possibly owing to its open access policy and broad research scope. Following closely is IEEE ACCESS, with 360 articles, showcasing the diversity and breadth of the field. SCIENTIFIC REPORTS ranks third with 358 articles, highlighting the intersection of medical AI. The JOURNAL OF MEDICAL INTERNET RESEARCH, with 347 articles, likely covers topics related to sensor technology and its applications in medical AI. Additionally, the JOURNAL OF THE AMERICAN MEDICAL INFORMATICS ASSOCIATION and SENSORS hold significant influence in the medical informatics field with 347 and 338 articles, respectively. The publication volume of these journals reflects the continued growth and interdisciplinary nature of the medical artificial intelligence field. Other journals such as APPLIED SCIENCES-BASEL, JMIR MEDICAL INFORMATICS, and BMC MEDICAL INFORMATICS AND DECISION MAKING also make substantial contributions, demonstrating the diversity and activity within the field of medical artificial intelligence.

In 2023, bibliometric analysis of medical artificial intelligence research unveiled several highly cited global publications. Leading the list is the review article titled "ChatGPT Utility in Healthcare Education, Research, and Practice: Systematic Review on the Promising Perspectives and Valid Concerns," published in the journal "Healthcare," which garnered 332 citations (21). Following closely is the Original Investigation article "Comparing Physician and Artificial Intelligence Chatbot Responses to Patient Questions Posted to a Public Social Media Forum," published in JAMA Internal Medicine, with 238 citations (22). Additionally, a review from Cureus titled "Artificial Hallucinations in ChatGPT: Implications in Scientific Writing" ranks as the third most cited (23), while an article from the Journal of Medical Systems titled "Evaluating the Feasibility of ChatGPT in Healthcare: An Analysis of Multiple Clinical and Research Scenarios" holds the fourth position (24). Notably, the top five most cited publications all center around topics related to ChatGPT.

### 5.4 Documents

Table 1 presents an overview of the top ten most globally cited reviews and articles within the field of medical artificial intelligence. The most globally cited review, "Machine learning: Trends, perspectives, and prospects," authored by M. I. Jordan from the University of California and T. M. Mitchell from Carnegie Mellon University, was published in the Science journal on July 17, 2015 (25). The top three most globally cited articles in the field of medical artificial intelligence literature are as follows: Guo L’s article titled "A multi-time scale approach to remaining useful life prediction in rolling bearing," published in Neurocomputing in 2017, investigates a multi-time scale modeling approach for predicting the remaining useful life (RUL) of rolling bearings. This study contributes to the advancement of predictive maintenance techniques in industrial applications (26). The second article, authored by A. Bate and titled "A Bayesian neural network method for adverse drug reaction signal generation," was published in the European Journal of Clinical Pharmacology in 1998 and has accumulated 692 citations globally (27). This article suggests that Bayesian neural network models can effectively detect significant signals from adverse drug reaction data, particularly from the WHO Programme on International Drug Monitoring dataset. Lastly, Wang LD’s research, titled "COVID-Net: a tailored deep convolutional neural network design for the detection of COVID-19 cases from chest X-ray images," stands out with 690 citations worldwide (28).

### 5.5 Topics

From Figure 4, it can be seen that these Trend Topics were calculated using keywords plus from the literature. We observed several important themes. Among them, neural networks have received widespread attention in this field, appearing 11 times with a time span from 2001 to 2020, showing a gradually increasing trend. Clinical medicine is also one of the research hotspots, appearing 13 times, covering the period from 1998 to 2011, indicating long-term research attention. Research on medical records has also been highly regarded, appearing as many as 181 times, spanning from 2014 to 2021, and showing an increasing trend year by year, demonstrating the emphasis on medical information management. Decision support systems also play an important role in the field of medical artificial intelligence, appearing 52 times from 2012 to 2022, indicating sustained research interest. In addition, topics such as primary care, forecasting, models, and health have also received widespread attention, with research frequencies of 151, 1607, 1248, and 1230 times respectively, showing a continuous growth trend in recent years. Additionally, emerging topics such as COVID-19 and drug discovery have also attracted considerable research attention in a short period, indicating the rapid response and adaptability of the field of medical artificial intelligence to current important issues.

The bibliometric analysis of national cooperation depicted in this graph illustrates the collaborative research endeavors among different countries. The size of nodes in the graph represents the research output (number of published papers) of each country, while the connections between nodes indicate collaborative relationships, with thicker lines denoting closer cooperation. From this graph, it can be observed that China stands out as the leading contributor in artificial intelligence in health care research, demonstrating both prolific research output and frequent collaboration with other countries, thus showcasing its prominent position in international research collaboration. Additionally, countries like the United States (unlabeled), Japan, and South Africa exhibit strong inclinations toward collaboration.

### 5.6 Co-occurrences

The analysis of co-occurrence networks based on titles in bibliometrics is a method used to extract keywords from a large number of document titles and construct co-occurrence networks based on these keywords, revealing the associations and connections among documents. Our study indicates that after categorizing the extracted titles, they can be classified into five groups (Figure 5). Cluster 1 focuses on the application of neural networks in medical imaging, including terms like "neural," "network," "luster underscores the use of deep learning techniques for tasks such as disease detection and classification. Cluster 2 revolves around healthcare information systems and patient data management, featuring terms like "health," "electronic record," "care," "systems," and "patient." It highlights the significance of electronic health records and data systems in healthcare analytics and decision-making. Cluster 3 delves into machine learning, predictive modeling, and various medical applications, encompassing terms like "machine learning," "prediction," "analysis," "model," "covid," "disease," "cancer," "medical," "application," and "techniques." This cluster indicates a broad exploration of machine learning methodologies for predictive modeling and analysis in healthcare settings. Cluster 4 centers on artificial intelligence and its applications in medicine, including terms like "artificial intelligence," "review," "medicine," "applications," and "systematic." It suggests a focus on systematic reviews and applications of artificial intelligence in healthcare research and practice. Cluster 5 covers clinical research, patient studies, and risk assessment, featuring terms like "data," "study," "patients," "clinical," "system," "risk," "development," and "assessment." This cluster highlights the use of data-driven approaches for clinical research, patient management, and risk assessment in healthcare settings.

## 6 Discussion

Our analysis reveals the research landscape of the past 30 years in the field of medical artificial intelligence. The remarkable expansion of scientific productivity from 2019 to 2023 is particularly noteworthy, reflecting an unprecedented period of growth and innovation in the field of bibliometrics in recent years. In recent years, with the advancement of deep learning, machine learning algorithms, hardware capabilities, and databases, artificial intelligence technology has experienced a third wave of development, providing powerful assistance to clinical work (24). As evidenced by our research findings, there has been a growing trend in artificial intelligence research in the medical field, from only 15 publications in 1995 to 310 publications in 2015, and reaching 5,297 publications by 2023.

The United States leads in publications, followed closely by China, with India ranking third. The United States holds a significant lead in medical artificial intelligence for several key reasons: 1) The U.S. healthcare industry generates a vast amount of diverse medical data, providing a rich environment for the application of artificial intelligence and machine learning technologies. 2) Major U.S. tech companies such as Google, IBM, and Amazon have been heavily investing in and applying their expertise in artificial intelligence to the healthcare sector, developing tools for medical imaging analysis, drug discovery, and virtual healthcare. 3) Despite ongoing developments in its regulatory environment, the United States exhibits a more lenient attitude toward the adoption of artificial intelligence in healthcare compared to other countries, leading to substantial private investments in medical artificial intelligence startups. In contrast, the impact of the EU AI Act on innovation is complex, involving legal, policy, and social dimensions, which makes it challenging to draw definitive conclusions within the scope of this study. As a result, existing literature on the topic is presented without an in-depth exploration of the Act’s. 4) The U.S. government allocates funding for medical artificial intelligence research through various institutions such as the National Institutes of Health and the Food and Drug Administration, supporting research in areas such as medical imaging analysis, disease diagnosis, and drug discovery. Moreover, China, with a higher proportion of international collaboration than the United States, actively promotes the development and application of artificial intelligence through measures such as formulating development plans, driving technological innovation, strengthening patent applications, promoting industrial ecosystem construction, and proposing global governance initiatives. Patent applications, as an early indicator of technological trends and innovation, allow for timely identification of emerging topics and standardized cross-national comparisons, avoiding biases introduced by varying patent approval times across countries. China leads the world in the number of patent applications related to artificial intelligence, reflecting its innovative vitality in this field. From January 2018 to October 2022, China filed over 648,000 patent applications related to artificial intelligence, far surpassing the numbers from the United States and South Korea (29).

As our research indicates, over the past 20 years, around 2002, the development and application of neural networks became one of the focal points in medical artificial intelligence research. At that time, the potential importance of artificial intelligence technologies, particularly neural networks, in providing innovative solutions for tasks such as medical imaging analysis, disease diagnosis, and personalized treatment recommendations, was already evident (30–34). Furthermore, the rapid growth of emerging topics such as COVID-19 in recent years is also worth noting. COVID-19, as a global public health crisis, has spurred interest in pandemic management and treatment within the medical artificial intelligence field. Researchers have utilized artificial intelligence technologies for COVID-19 prediction, virus tracing, drug discovery, and other aspects to address this challenge (35–39). Particularly in drug discovery, artificial intelligence technologies such as deep learning and machine learning have been widely applied, accelerating the drug development process (40–43). Additionally, the application of blockchain technology in the field of medical artificial intelligence has garnered significant attention (44, 45). The decentralized, tamper-proof, and secure characteristics of blockchain provide new solutions for medical data management, medical information sharing, and medical privacy protection (46–49). Therefore, the emergence of COVID-19, drug discovery, blockchain, and other emerging topics enriches the research landscape of medical artificial intelligence, expands its application scope, and holds significant implications for promoting medical innovation and addressing practical issues.

When examining the most cited literature in our study, we surprisingly found that all five of the highest cited publications in 2023 were related to medical artificial intelligence research involving ChatGPT. This indicates the widespread attention and citation of ChatGPT’s application and research in the medical field, demonstrating the importance and influence of artificial intelligence in medicine. Current research has shown that ChatGPT has a wide range of applications in medical research (19). This natural language processing-based artificial intelligence technology can assist physicians in better understanding patients’ conditions, bringing innovation to the medical field. In medical writing, ChatGPT is used to generate initial drafts of documents, assist medical authors with writing support, and automate the review and editing process (23). Additionally, ChatGPT can be applied to medical education to help adjust teaching strategies, prevent inappropriate use, and promote the cultivation of students’ critical thinking abilities (21).

The rapid advancement of artificial intelligence (AI) holds great promise for medical applications. Personalized medicine, integrating genomic and other patient data, is poised to enable precise diagnosis and tailored treatment. Interdisciplinary collaboration is driving innovation in areas such as intelligent diagnostic devices and drug delivery systems. Strengthening ethical and regulatory frameworks is critical to safeguarding privacy, fostering trust, and ensuring patient-centered AI development. Moreover, international cooperation can expand AI applications to resource-limited regions, enhancing healthcare accessibility and reducing disparities. Notably, ChatGPT has emerged as a transformative tool, optimizing patient consultation workflows, streamlining medical documentation, boosting research efficiency, and supporting personalized learning and simulation in medical education.

There are also limitations in this study. Firstly, many computer-related papers in medical artificial intelligence are often published in conference proceedings, which may lead to an underestimation of the impact of such research. Additionally, ChatGPT not only assists clinicians in better understanding patient conditions in clinical practice, but also provides new ideas and solutions in medical research. It is expected that significant changes will be observed in the bibliometric analysis of related literature in 2024.

## 7 Conclusion

Overall, the development of artificial intelligence (AI) in healthcare is closely linked to advancements in foundational AI techniques, with applications like ChatGPT showcasing this trend. AI’s integration into healthcare has addressed critical societal needs, particularly in responding to challenges such as COVID-19 and breakthrough drug discovery. While this study examines the evolution of AI in healthcare over the past three decades, certain areas, such as calculating the proportion of AI-related papers and exploring the relationship between impact factor and research quality, remain for future research. These aspects require careful planning and are beyond the current study’s scope, but they are crucial for further enriching our findings and understanding AI’s broader impact on healthcare. Future studies will aim to incorporate economic and population data and explore these relationships in greater depth, providing valuable insights for policymakers, researchers, and clinicians.

## Data Availability

The raw data supporting the conclusions of this article will be made available by the authors, without undue reservation.
